# Goal-Directed Processing of Naturalistic Stimuli Modulates Large-Scale Functional Connectivity

**DOI:** 10.3389/fnins.2018.01003

**Published:** 2019-01-29

**Authors:** Zhenfu Wen, Tianyou Yu, Xinbin Yang, Yuanqing Li

**Affiliations:** ^1^Center for Brain Computer Interfaces and Brain Information Processing, South China University of Technology, Guangzhou, China; ^2^Guangzhou Key Laboratory of Brain Computer Interaction and Application, Guangzhou, China; ^3^Department of Surgical Thoracic Oncology, Affiliated Cancer Hospital and Institute of Guangzhou Medical University, Guangzhou, China

**Keywords:** top-down goals, naturalistic condition, inter-subject functional correlation, multivariate pattern analysis, large-scale brain networks

## Abstract

Humans selectively process external information according to their internal goals. Previous studies have found that cortical activity and interactions between specific cortical areas such as frontal-parietal regions are modulated by behavioral goals. However, these results are largely based on simple stimuli and task rules in laboratory settings. Here, we investigated how top-down goals modulate whole-brain functional connectivity (FC) under naturalistic conditions. Analyses were conducted on a publicly available functional magnetic resonance imaging (fMRI) dataset (OpenfMRI database, accession number: ds000233) collected on twelve participants who made either behavioral or taxonomic judgments of behaving animals containing in naturalistic video clips. The task-evoked FC patterns of the participants were extracted using a novel inter-subject functional correlation (ISFC) method that increases the signal-to-noise ratio for detecting task-induced inter-regional correlation compared with standard FC analysis. Using multivariate pattern analysis (MVPA) methods, we successfully predicted the task goals of the participants with ISFC patterns but not with standard FC patterns, suggests that the ISFC method may be an efficient tool for exploring subtle network differences between brain states. We further examined the predictive power of several canonical brain networks and found that many within-network and across-network ISFC measures supported task goals classification. Our findings suggest that goal-directed processing of naturalistic stimuli systematically modulates large-scale brain networks but is not limited to the local neural activity or connectivity of specific regions.

## 1. Introduction

Selective processing of information according to behavioral goals is crucial for our interaction with the complex environment. However, the organizational basis underlying this goal-directed behavior is unclear. Electrophysiological and functional imaging studies have suggested that task goals modulate the neural representation of a stimulus (Mirabella et al., 2007; Ptak and Schnider, 2010; Gilbert and Li, 2013). Recently, using powerful multivariate pattern analysis (MVPA) methods (Norman et al., 2006; Haxby et al., 2014), many studies have found that top-down behavioral goals can be decoded with distributed activities across frontoparietal and sensory regions (Chiu et al., 2011; Waskom et al., 2014; Loose et al., 2017; Long and Kuhl, 2018).

Despite numerous studies, most neural investigations of goal-directed behavior have employed simple stimuli, such as moving dots, static faces and object images, which are functionally well-characterized (Nastase et al., 2017). However, many common perceptual tasks require combining low-level features of stimuli to represent abstract semantic information in our brains. With the recent observation that less-controlled naturalistic stimuli such as movies evoke reliable neural responses across individuals (Hasson et al., 2004, 2010; Simony et al., 2016), a few studies have used natural paradigms to investigate how task contexts modulate the neural representation of high-level visual and semantic information (Cukur et al., 2013; Nastase et al., 2017, 2018). These pioneering studies have found that goal-directed processing of different objects or semantic features in natural movies modulates distributed cortical areas. However, these studies mainly focused on the modulation effects of behavioral goals on neural activity in certain brain regions but ignored the interaction between distributed cortical areas, which are increasingly recognized as the biological basis for cognition and behaviors (Fox et al., 2005; Sporns, 2014; Mišić and Sporns, 2016).

Evidence from electrophysiology studies indicates the important role of neuronal synchronization in goal-directed behavior (Von Stein and Sarnthein, 2000; Engel et al., 2001; Womelsdorf et al., 2007). Similar findings have also been reported in recent functional magnetic resonance imaging (fMRI) studies (Spreng et al., 2010; Al-Aidroos et al., 2012). For example, activity in V4 is correlated more strongly with activity in the fusiform face area in a face attention task and with activity in the parahippocampal place area in a scene attention task (Al-Aidroos et al., 2012). Notably, most of these studies used rudimentary visual stimuli and primarily focused on connections between a limited number of brain regions that were selected based on prior anatomical knowledge or on activation patterns during tasks. These preselecting methods may lead to some regions being ignored, given that goal-directed processing of external information recruits a wide variety of brain regions (Corbetta and Shulman, 2002; Petersen and Posner, 2012; Vaziripashkam and Xu, 2017). Accordingly, examining whole-brain functional connectivity (FC) may provide new insights into top-down goals representation.

A standard method to characterize whole-brain FC is to calculate a Pearson correlation between the time series of all pairs of regions within each subject (van den Heuvel and Hulshoff Pol, 2010; Zalesky et al., 2012). However, a potential limitation of this method is that the calculated FC measures consist of task-evoked correlations, within-subject intrinsic neural fluctuations, and non-neuronal artifacts, and these types of signals cannot be reliably separated (Hasson et al., 2004; Simony et al., 2016). Given that the FC structure during task performance has been shown to be highly correlated with the intrinsic FC structure (Cole et al., 2014), it would be difficult to reliably detect differences in FC patterns across task contexts. Recently, a novel method termed inter-subject functional correlation (ISFC) has been proposed (Simony et al., 2016). By calculating inter-regional correlations between subjects that are performing the same task, the ISFC method increases the signal-to-noise ratio (SNR) for detecting task-evoked FC, making it an effective method for examining subtle differences between cognitive states (Simony et al., 2016; Rosenthal et al., 2017).

In the present study, we applied the ISFC method to a publicly available dataset to investigate how behavioral goals modulate whole-brain FC. Dynamic video clips of animals behaving in natural environments were used as stimuli. During the fMRI experiment, participants were required to made either behavioral or taxonomic judgments when exposed to identical naturalistic video clips. We used MVPA methods to explore task modulation of whole-brain FC. We show that ISFC patterns support successful task classification and that task goals modulate connections between large-scale brain regions that can be assigned to a variety of canonical functional networks.

## 2. Materials and Methods

### 2.1. Subjects

A publicly available dataset was used in this study (Nastase et al., 2017, 2018). This dataset was obtained from the OpenfMRI database (http://www.openfmri.org), and the accession number was ds000233. A total of 12 right-handed healthy adults (7 females; mean age = 25.4 ± 2.6 SD years) provided informed consent and participated in the main experiment. The study was approved by the Institutional Review Board of Dartmouth College.

### 2.2. Experimental Design

The experimental paradigm was described clearly in the original paper of Nastase et al. (2017, 2018). We briefly describe the most relevant aspects of the experimental design here for completeness. A total of 80 naturalistic clips of behaving animals (each lasting 2 s), collected from the Internet, were used in the experiment. Semantically, these clips could be partitioned into five groups based on taxonomic categories (primates, ungulates, birds, reptiles, and insects) or four groups based on behavioral categories (eating, fighting, running, and swimming). Each participant completed 10 experimental runs (each lasting 392 s) while viewing these clips under two task contexts. In half of the runs, participants were instructed to pay attention to taxonomy types in the presented clips (taxonomy task runs), and in the other half of the runs, participants were instructed to pay attention to the behavioral types of the stimuli (behavior task runs). These 5 taxonomic attention runs and 5 behavior attention runs were presented in a counterbalanced order across participants. Note that the appearance order of movie clips in each experimental run of each subject was randomized. Therefore, the appearance orders of clips in the two tasks were irregular, it is unlikely that the following MVPA results were contributed by differences of stimulus sequences between the two tasks.

In the taxonomy task runs, participants were asked to press a button if two sequential clips contained the same taxonomic category. In the behavior task runs, participants were asked to press a button if two sequential trials contained the same behavioral category. There were 4 repetition trials in each run that required a response. These tasks required participants to attend to the taxonomic or behavioral features of clips in a corresponding task context.

### 2.3. Image Acquisition

Functional and structural images were acquired on a 3 T Philips Intera Achieva MRI scanner with a 32-channel head coil. Functional images were obtained using single-shot gradient-echo echo-planar imaging with a SENSE reduction factor of 2 (TR/TE = 2,000/35 ms, flip angle = 90°, resolution = 3 mm isotropic, matrix size = 80 × 80, FOV = 240 × 240 *mm*^2^, 42 transverse slices in an interleaved fashion). Each participant completed 10 experimental runs in a scanning session, with an additional structural scan obtained at the end of the session using a high-resolution T1-weighted 3D turbofield echo sequence (TR/TE = 8.2/3.7 ms, flip angle = 8°, resolution = 0.938 × 0.938 × 1.0 *mm*^3^, matrix size = 256 × 256 × 220, FOV = 240 × 240 × 220 *mm*^3^).

### 2.4. Image Preprocessing

Imaging data were preprocessed using SPM12 (http://www.fil.ion.ucl.ac.uk/spm) and DPARSF (Chao-Gan and Yu-Feng, 2010). Functional data were slice-time adjusted, motion-corrected, and normalized to the Montreal Neurological Institute (MNI) space using a segmented high-resolution gray matter structural image and a gray matter template. The resulting images were detrended to abandon linear trends. The nuisance time series, including motion, white matter, CSF and their derivatives, were regressed out using linear regressions. Low-frequency signals were removed using a high-pass filter (>0.08 Hz). We did not use a low-pass filter, as in resting-state fMRI studies, as this allowed us to retain potentially informative task signals at higher frequencies (Shirer et al., 2012; Cole et al., 2013). Signals corresponding to stimulus presentation were further removed using standard general linear regression models of task events (Cao et al., 2014; Cole et al., 2014). Specifically, task events were modeled by convolving stimulus onsets with the standard hemodynamic response function. These regressors were then regressed out from voxel activities. The resultant residual time series were used for the following functional network analyses.

### 2.5. Definition of Nodes

A 264-node brain atlas was used for FC analysis. This atlas was derived from both resting and task FC meta-analyses (Power et al., 2011) and has been widely used in network analyses (Vatansever et al., 2015; Schultz and Cole, 2016). Each of the 264 nodes was assigned to one of the 14 subnetworks in the original publication (Cole et al., 2013). Among these 14 subnetworks, we focused on 10 well-established subnetworks, including the frontoparietal, cingulo-opercular, salience, dorsal attention, ventral attention, default mode, somatomotor (hand and mouth), auditory, visual, and subcortical networks. The other three networks, including the cerebellum network, the memory retrieval network, and a network of uncertain function, were also involved in our analyses, but they were treated as a single subnetwork (the others network) for convenience. Therefore, the 264 nodes were assigned to 11 subnetworks in this study. The nodal-mean time series were extracted by averaging the time series over all voxels in each of the 264 nodes, resulting in a neural signal matrix *X*, which has the form of a *P* × *N* matrix containing time series from *P* nodes over *N* time points. The neural signal matrix of each subject and each experimental run was used for the following network constructions.

### 2.6. Inter-subject Functional Correlation

We used the recently proposed ISFC to assess task-evoked FC (Simony et al., 2016). The ISFC method effectively eliminates intrinsic signals by calculating the inter-regional correlations between different subjects who perform the same task. Assuming we have a neural signal matrix *X*_*k*_ for each subject *k, k* = 1, …, *K* with each regional time series normalized to a zero mean and unit variance. In contrast to the standard FC measure, which is calculated within each neural signal matrix, the ISFC of subject *k* is defined as the Pearson correlation between this subject and the average of all other subjects:

(1) $${\overset{̭}{C}}_{k}=\frac{1}{N}X_{k}\left[\frac{1}{K-1}\sum_{q\neq k}X_{q}^{T}\right]$$

which is a *P* × *P* correlation matrix where each element (*i, j*) represents a correlation between node *i* of subject *k* and the mean series of node *j* of the other subjects. To increase the normality of the distribution of correlation values, each correlation coefficient was converted to a z-score using Fisher's *r*-to-*z* transformation. To further impose symmetry, the final ISFC matrix of subject *k* was given by $\left({\overset{̭}{C}}_{k}+{\overset{̭}{C}}_{k}^{T}\right)/2$. The group-based ISFC matrix was calculated by averaging the ISFC matrixes across subjects:

(2) $$\overset{̭}{C}=\frac{1}{K}\sum_{k}{\overset{̭}{C}}_{k}$$

### 2.7. Similarity Analysis

A key question of this study was whether goal-directed visual processing modulates whole-brain FC. Conceptually, if the task goal modulates ISFC, similarities between ISFC patterns from the same task should be higher than those from different tasks. To confirm this hypothesis, we performed a similarity analysis as follows. First, the neural signal matrices of each subject were averaged across the 5 behavioral task runs and the 5 taxonomic task runs, resulting in two neural signal matrices (one for the behavioral task and one for the taxonomic task) for ISFC pattern estimations. Then, the 12 subjects were randomly split into two independent groups of 6 subjects, and group-based ISFC matrices were calculated for each group and each task according to Equation (2). Next, we calculated the between-task ISFC similarity and within-task ISFC similarity across the two groups. Specifically, the between-task ISFC similarity was defined as the spatial Pearson correlation between the ISFC matrices from different groups and different tasks (e.g., group 1 task 1 vs. group 2 task 2). The within-task ISFC similarity was defined as the spatial Pearson correlation between the ISFC matrices from different groups and the same task (e.g., group 1 task 1 vs. group 2 task 1). We repeated this procedure 462 times (all possible situations with the 12 subjects divided into two groups of 6 subjects) and compared the mean within-task ISFC similarities and mean between-task ISFC similarities across all situations.

### 2.8. ISFC Classification of Attention Task

We further used MVPA methods to examine whether the task goals of subjects could be predicted using whole-brain ISFC patterns. A leave-one-subject-out-cross-validation (LOSOCV) procedure was employed to assess the classification performance. In each iteration of the LOSOCV, we left out the data of one subject as the test set and use the data of the other subjects as the training set. A template-matching method was used for task label predictions (Simony et al., 2016). Similar to the similarity analysis, neural signal matrices were first averaged across tasks for each subject within the training set. Then, the group-based ISFC matrices were calculated according to Equation (2) for each attention task based on the corresponding neural signal matrices. Therefore, based on the training data, we obtained one ISFC matrix, *C*_*beh*_, for the behavioral task and one ISFC matrix, *C*_*tax*_, for the taxonomic task. These two matrices were used as ISFC templates for the two attention tasks. Note that the test data were never used to derive the ISFC templates.

For each run *r* of the left-out subject, we attempted to predict its task label by comparing its ISFC matrix with the two ISFC templates. We calculated an ISFC matrix, *C*_*beh, r*_, between the run r and the average neural signal matrix corresponding to the behavior task from the training set. Similarly, we also obtained an ISFC matrix, *C*_*tax, r*_, for the taxonomic task. The predicted label for this run was then given by the attention task *m*∈{*beh, tax*} that maximized the Pearson correlation between *C*_*m, r*_ and the templates *C*_*m*_:

(3) $${\overset{̭}{m}}_{r}=\underset{m\in \{beh,tax\}}{\text{arg min}}\{Corr(C_{m,r},C_{m})\}$$

This procedure was repeated for each subject and each run, and the classification accuracy was then computed as the proportion of times that an experimental run was assigned to the correct task context.

### 2.9. FC Classification of Attention Task

For comparison, we also used standard FC to classify task goals. This procedure was similar as the ISFC classification procedure described above except the FC matrixes were calculated within subjects. We obtained a correlation matrix for each subject and each experimental run by calculating the Pearson correlation coefficient between every pair of nodes. The correlation matrixes of the same task were further averaged within each subject. Averaging correlation matrixes of each task increased the signal-to-noise ratio (SNR) of estimated FC templates. Previous MVPA studies have suggested that this average step often improve classification performance to some degree (Isik et al., 2013; Hebart et al., 2018). For the employed template-matching method in this study, the testing sample was assigned the label of the FC template with which it is maximally correlated. Therefore, we would expect a better classification performance by increasing the SNR of estimated FC templates. Then, using training data, we obtained two FC templates *C*_*beh*_ and *C*_*tax*_ by averaging the correlation matrixes of the behavioral tasks and taxonomic tasks across subjects, respectively. For a run *r* of the test subject, we obtained its within-subject FC matrix *C*_*r*_, and the label of this FC matrix was predicted as the task that maximized the Pearson correlation between *C*_*r*_ and the templates *C*_*m*_:

(4) $${\overset{̭}{m}}_{r}=\underset{m\in \{beh,tax\}}{\text{arg min}}\{Corr(C_{r},C_{m})\}$$

### 2.10. Identifying Discriminative Connections

Connections contribute differently to classification. To determine discriminative connections that contributed more to task classification, we performed an edge-based analysis similar to a previous study (Finn et al., 2015). Computationally, the Pearson correlation of two normalized vectors (zero mean, unit variance) was calculated as the sum of the element-wise products. Thus, an element with a large positive product contributes more to the correlation coefficient. In this classification procedure, we calculated Pearson correlation coefficients between the ISFC matrix derived from the test data and the templates derived from the training data, and the task label was chosen as the one that resulted in the largest correlation coefficient. Conceptually, the product of a discriminative connection should be large when the test data and the template are from the same task. In contrast, the product should be small when the test data and the template are from different tasks. Therefore, given a test ISFC matrix *C*_*m, r*_ for run *r* and templates *C*_*m*_, *m* ∈ {*beh, tax*}, we defined the discriminative measure of edge *e* as:

(5) $$\begin{array}{r} \phi_{m,r}\left(e\right)=Comp\left(C_{m}\left(e\right)*C_{m,r}\left(e\right),C_{u}\left(e\right)*C_{m,r}\left(e\right)\right)\;u\in \left\{beh,tax\right\},u\neq m \end{array}$$

where *Comp*(*a, b*) = 1 if *a* > *b*, otherwise *Comp*(*a, b*) = 0. The first term of the function, *Comp*(), is the within-task edge-wise product, and the second term is the between-task edge-wise product. The discriminative measures were then averaged across all iterations of the LOSOCV to obtain a single value, ϕ(*e*), for each connection *e*. A connection with a large ϕ(*e*) is thought to be discriminative.

### 2.11. Subnetwork-Based Classification

To further assess the classification ability of individual canonical subnetworks, we conducted the same LOSOCV procedure as described above. However, this time, only within-network connections calculated between regions from a specific subnetwork or across-network connections calculated between regions from two different canonical subnetworks were used for task classification.

### 2.12. Effects of Scan Length on Classification

Since an experimental run consisted of a relatively long time series (196 time points), we further explored whether task goals could be predicted using fewer time points. We varied the number of time points n that were used to calculate the ISFC measures between 20 and 180 in increments of 10. Following previous studies (Finn et al., 2015; Greene et al., 2018), for each number of time points n, we randomly chose the start time point, and then extracted n continues time points beginning with that starting point to calculate the ISFC measures for task classification. We did not extract time points randomly from the whole series, because this strategy may ignore the temporal autocorrelation of fMRI time series (Woolrich et al., 2001) and thus bias the estimation of functional connectivity. This procedure was repeated 10 times, and the mean classification accuracies for these times were obtained.

### 2.13. Effects of Atlas on Classification

To test whether the task classification accuracy based on ISFC measures was sensitive to the specific choice of atlas and network assignments, we conducted the aforementioned classification analyses using an additional 268-node atlas provided by Shen et al. (2010). This atlas functionally divides the brain into 268 regions by maximizing the similarities of the voxel-wise time series within each node and assigns each region to one of the following subnetworks: subcortical-cerebellum, frontoparietal, default mode, medial frontal, motor, visual 1, visual 2, or visual association.

### 2.14. Statistical Analysis

We used a non-parametric permutation test (Nichols and Holmes, 2002) to assess whether the difference between the mean within-task similarity and the mean between-task similarity was significant. We first combined the 462 within-task similarities and the 462 between-task similarities into one group. Then, we randomly split the group into two equal-size groups and calculated the difference between the means of these two groups. We repeated this procedure 1,000 times and obtained a null distribution of the differences. The *p*-value was then calculated as the number of null-hypothesis differences that were equal to or greater than the observed true difference divided by 1,000. With this approach, the smallest *p*-value that can be reported is 1/1, 000 = 0.001.

A non-parametric permutation test was also used to assess the statistical significance of the task classification accuracy. In brief, we first shuffled the labels of all the experimental runs and then performed the aforementioned LOSOCV procedure for ISFC features to obtain a classification accuracy. We repeated this procedure 1,000 times, resulting in a null distribution of accuracies. The *p*-values were calculated as described above.

For subnetwork-based classifications, to control for the presence of multiple comparisons (Nichols and Holmes, 2002), we obtained the maximum classification accuracy across all subnetworks in each iteration of the permutation. These maximum values were used to construct the null distribution of accuracies. Similarly, for classifications based on variations in scan length, the maximum accuracies for each assessed time point were used to construct a null distribution.

To determine the discriminative connections, we obtained the maximum discriminative measure for all connections at each iteration of the permutation. The null distribution was constructed using these maximum discriminative measures. Connections with true discriminative measures larger than the 95*th* percentile of the null distribution were considered to be discriminative (i.e., *p* < 0.05).

## 3. Results

### 3.1. Behavioral Results

As stated in the original publication of the dataset (Nastase et al., 2017), participants performed very well in both the behavior task (mean accuracy: 0.994, SD: 0.005) and the taxonomy task (mean accuracy: 0.993, SD: 0.005). A paired t-test revealed no significant task-related difference in detection accuracy [*t*_(11)_ = 0.469, *p* = 0.91]. In addition, response times were also not significantly different between the two conditions [paired *t*-test: *t*_(11)_ = 0.015, *p* = 0.99), though the small number of response trials might hinder a robust estimation of response times. Therefore, it is unlikely that the subsequent classification analyses were influenced by differential behavioral responses.

### 3.2. Modulation of Whole-Brain FC

**Figure 1:**
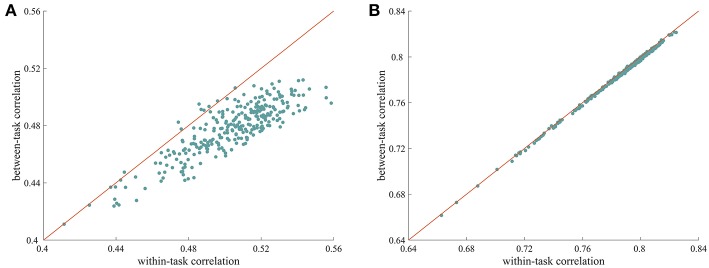
Modulation of whole-brain FC under different tasks. **(A)** Scatter plot of within-task correlation values vs. between-task correlation values calculated using ISFC patterns. The within-task correlation value was significantly higher than the between-task correlation value (*p* = 0.001, permutation test), indicating that ISFC patterns are more similar when subjects perform the same task than when they perform different tasks. **(B)** Scatter plot of within-task correlation values vs. between-task correlation values calculated using standard FC patterns. The two types of measures were highly correlated, with no significant differences observed (*p* = 0.63), suggesting that standard FC patterns are less sensitive at detecting modulated connections. Each data point corresponds to a random partition of the subjects.

We used a similarity analysis to examine the top-down modulation of whole-brain FC. As shown in Figure 1A, when the ISFC method was used to extract task-evoked FC patterns, the within-task similarities were larger than the between-task similarities, with most of the data points falling below the diagonal. This difference was significant, as indicated by a permutation test (*p* = 0.001). This finding indicates that whole-brain FC was modulated when humans processed the same stimulus with different task goals. In contrast, the standard FC method resulted in very similar values for within-task similarity and between-task similarity (Figure 1B), with no significant difference observed between the two types of measures (*p* = 0.19). This result is consistent with recent findings that connections observed during different tasks are highly correlated (Cole et al., 2014). The successful detection of attentional modulation of FC using the ISFC method may be attributed to the effectiveness of the ISFC method in eliminating intrinsic signals (Simony et al., 2016; Kim et al., 2017).

### 3.3. Classification of Task Contexts

**Figure 2:**
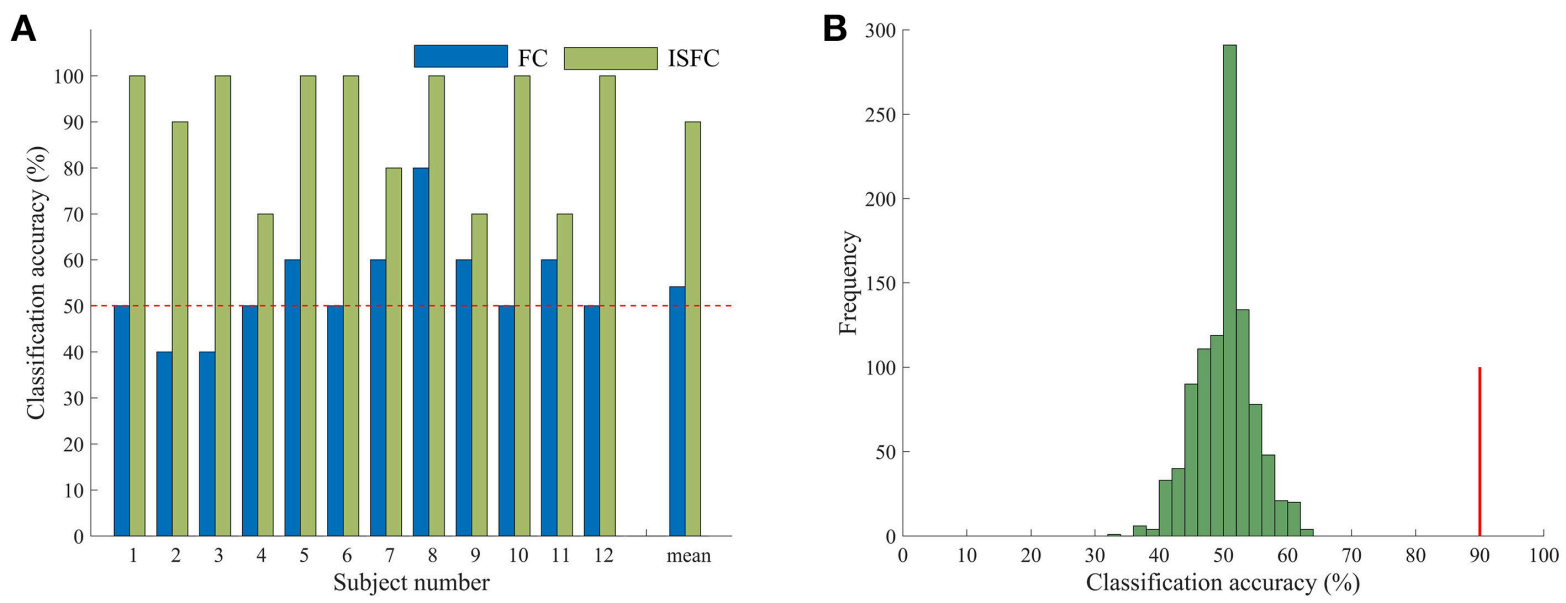
Accuracy in task classification. **(A)** Accuracy of each single subject and mean accuracy across the LOSOCV using ISFC and FC patterns. The horizontal dashed line shows the chance level (50%). **(B)** Permutation test of accuracy when ISFC patterns were used for classification. The histogram shows the null distribution of accuracy values when task labels were randomly permuted, and the solid red line indicates the accuracy obtained for the true task labels. The classification accuracy (90%) was significantly higher than the chance level (*p* = 0.001).

Having confirmed that task contexts modulated whole-brain FC, we further applied MVPA methods to investigate the possibility of task goals prediction. We used the ISFC method to extract task-evoked FC for each of the tasks and used a template-matching method to predict the task label of each experimental run of a left-out subject. The LOSOCV procedure showed an accuracy of 90% (Figure 2), which was significantly higher than chance (50%), as indicated by a permutation test (*p* = 0.001). We also performed the same LOSOCV procedure using standard FC patterns (Figure 2A). In this case, the accuracy was much lower and did not reach significance (54.17%, *p* = 0.13). Together with the similarity analyses, these results suggest that the ISFC method is powerful in detecting subtle differences between cognitive states. Since the classification accuracy was much higher with ISFC patterns than with standard FC patterns, we focused on ISFC patterns in the following analyses.

### 3.4. Connections Contribution to Classification

**Figure 3:**
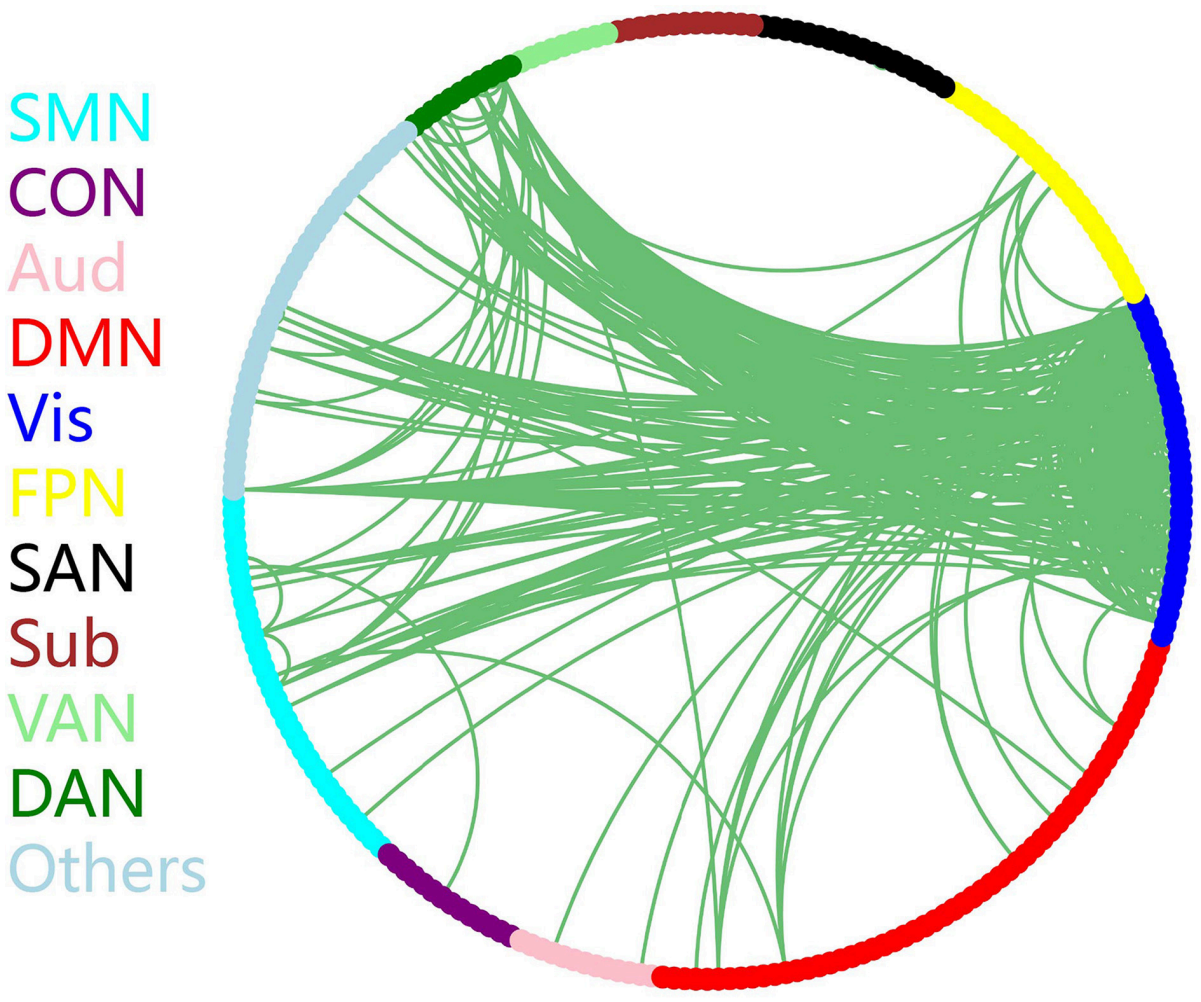
Distribution of discriminative connections identified by edge-wise analysis. Brain regions are arranged and color-coded according to 11 canonical subnetworks: frontoparietal (FPN), cingulo-opercular (CON), salience (SAN), dorsal attention (DAN), ventral attention (VAN), default mode (DMN), somatomotor (SMN), auditory (Aud), visual (Vis), subcortical (Sub), and a network with other regions (Others).

**Figure 4:**
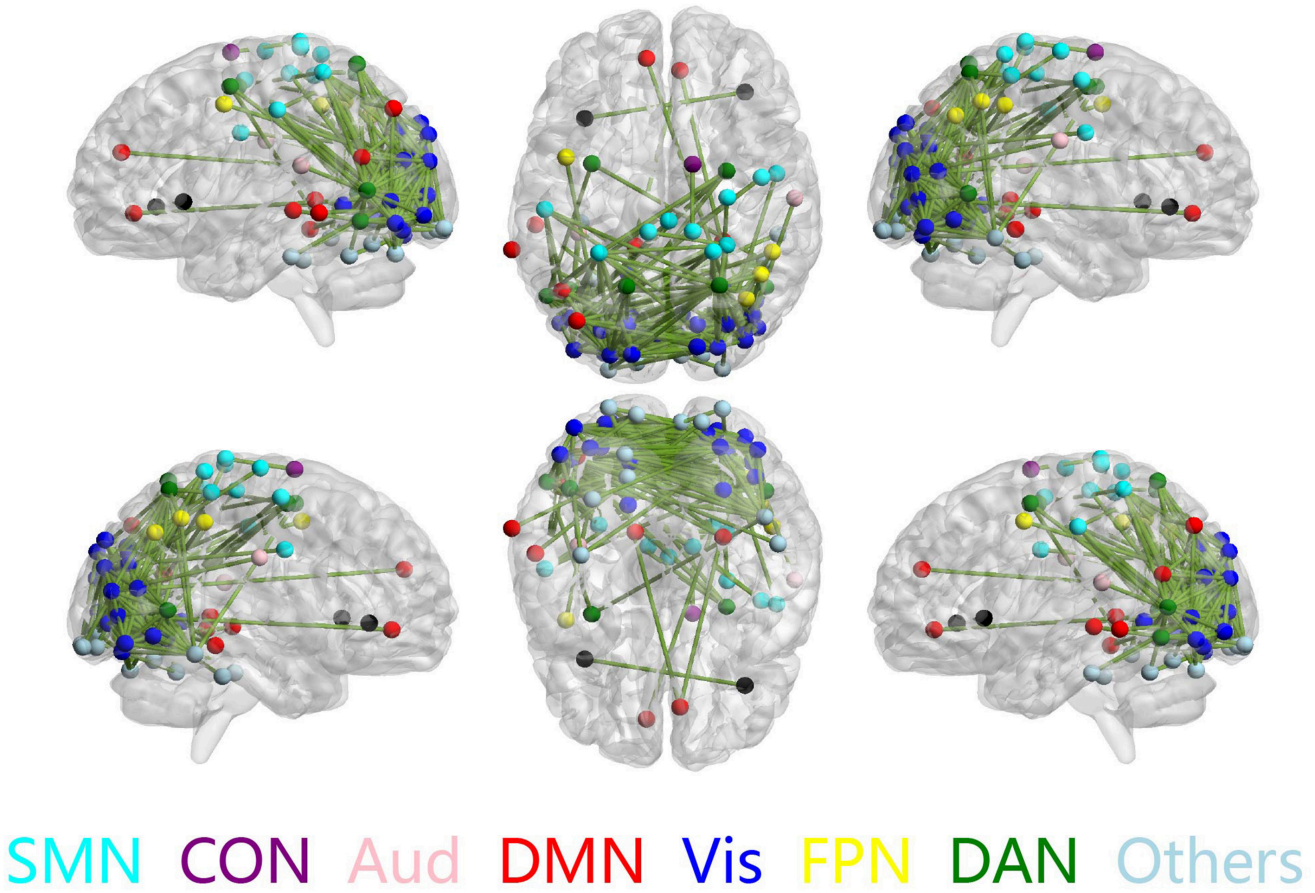
Discriminative connections shown in sagittal (left/right), axial (top/bottom), and coronal (front/back) views. Nodes indicate brain regions, and edges represent connections between regions. Only regions that formed discriminative connections are shown.

The discriminative connections that largely contributed to the classification were determined using edge-wise analysis. Specifically, for each edge, a mean discriminative measure was calculated across the LOSOCV procedure and compared to a null distribution constructed from 1,000 random permutations. We then identified discriminative edges as those that possessed discriminative measures larger than the 95th percentile of the null distribution. We found 383 discriminative connections among all 34980 possible connections. These discriminative connections are displayed in a circle plot (Figure 3) and projected to a surface rendering of a human brain (Figure 4) using the BrainNet viewer (Xia et al., 2013). The majority of the discriminative connections are within the visual network and between the visual network and other networks, mainly including the dorsal attention, somatomotor, and default mode networks.

### 3.5. Subnetwork-Based Classification

**Figure 5:**
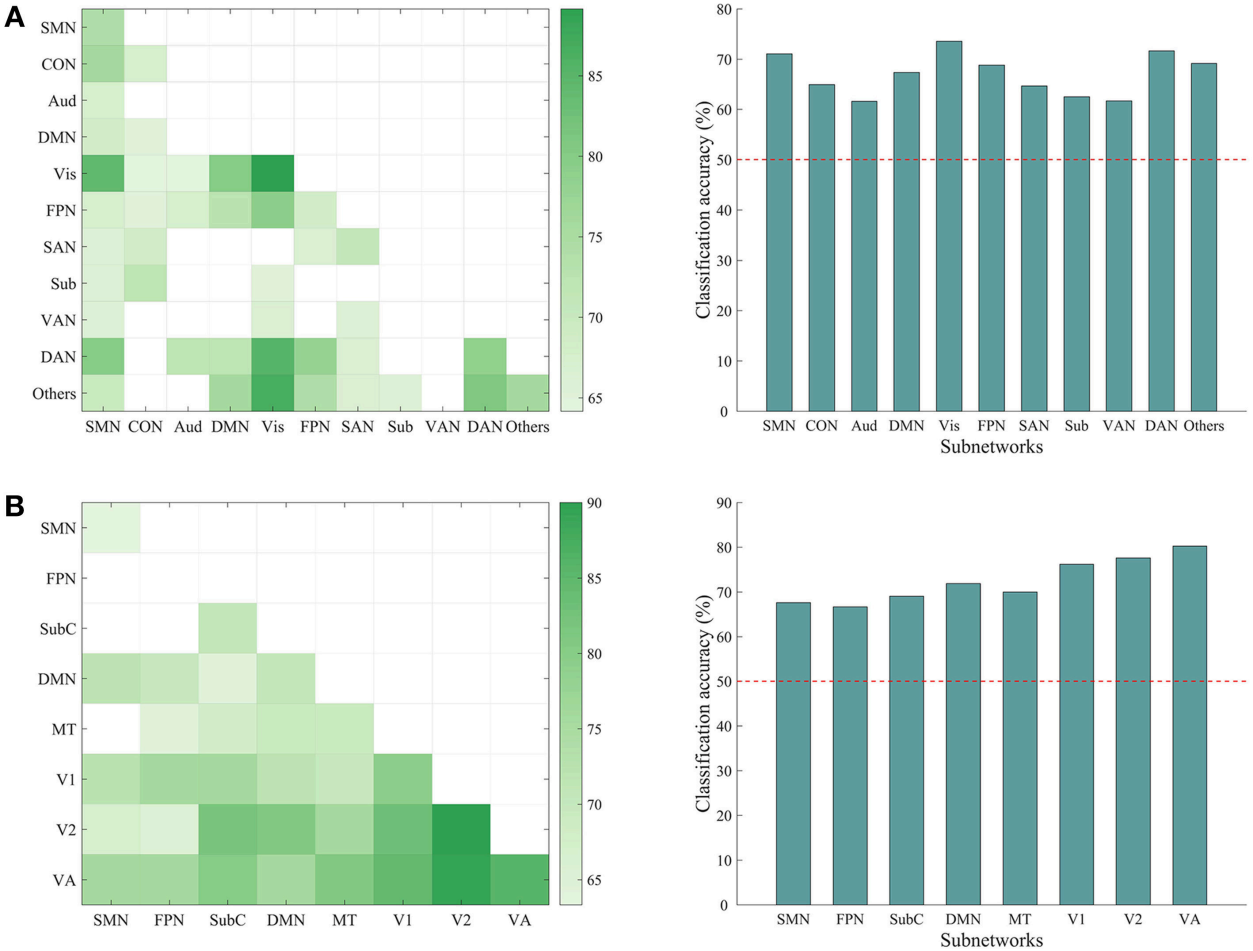
Classification accuracy using ISFC patterns of subnetworks. **(A)** Left: matrix representing the classification accuracy based on ISFC measures within and between canonical subnetworks. Rows and columns represent predefined subnetworks based on the 264-node atlas. Only accuracies significantly higher than the chance level (50%) are shown in the matrices (*p* < 0.05, corrected). The abbreviations for each subnetwork are the same as in Figure 3. Right: average accuracy based on across-network ISFC measures between a specific subnetwork on the x-axis and all the other subnetworks. The horizontal dashed line shows the chance level. Each of the subnetworks reached significance (all *p* = 0.001, corrected). **(B)** Similar information as presented in A, but with a different 268-node atlas that contained 8 subnetworks: medial frontal (SMN), frontoparietal (FPN), default mode (DMN), subcortical-cerebellum (SubC), motor (MT), visual 1 (V1), visual 2 (V2), and visual association (VA).

We have shown that the top-down behavior goals could be reliably classified using whole-brain ISFC patterns and found that discriminative connections were distributed across several networks. However, whether a specific subnetwork (e.g., the dorsal attention network) supports task classification was not clear. To explore this possibility, we performed classification analyses using the within-network ISFC measures from each of the 11 canonical networks separately. As shown in Figure 5, the classification accuracies of the somatomotor (74.16%, *p* = 0.001), cingulo-opercular (67.50%, *p* = 0.003), visual (89.170%, *p* = *0.001*), frontoparietal (68.33%, *p* = 0.001), salience (70.83%, *p* = *0.001*), dorsal attention (78.33%, *p* = 0.001) and the others network (75.83%, *p* = 0.001) were significantly higher than chance (50%).

We also tested whether the across-network ISFC measures between two subnetworks (e.g., connections between the visual and dorsal attention networks) would support the classification (Figure 5). This analysis revealed many discriminative across-network measures, mainly related to the visual, dorsal attention, frontoparietal, and somatomotor networks. To further assess the classification ability of each specific network, we averaged the accuracies obtained using across-network ISFC measures between each network and every other network. As shown in Figure 5, all across-network measures showed significant accuracies. These results suggest that the modulated connections are distributed across the brain and not limited to specific subnetworks.

### 3.6. Effects of Scan Length on Classification

**Figure 6:**
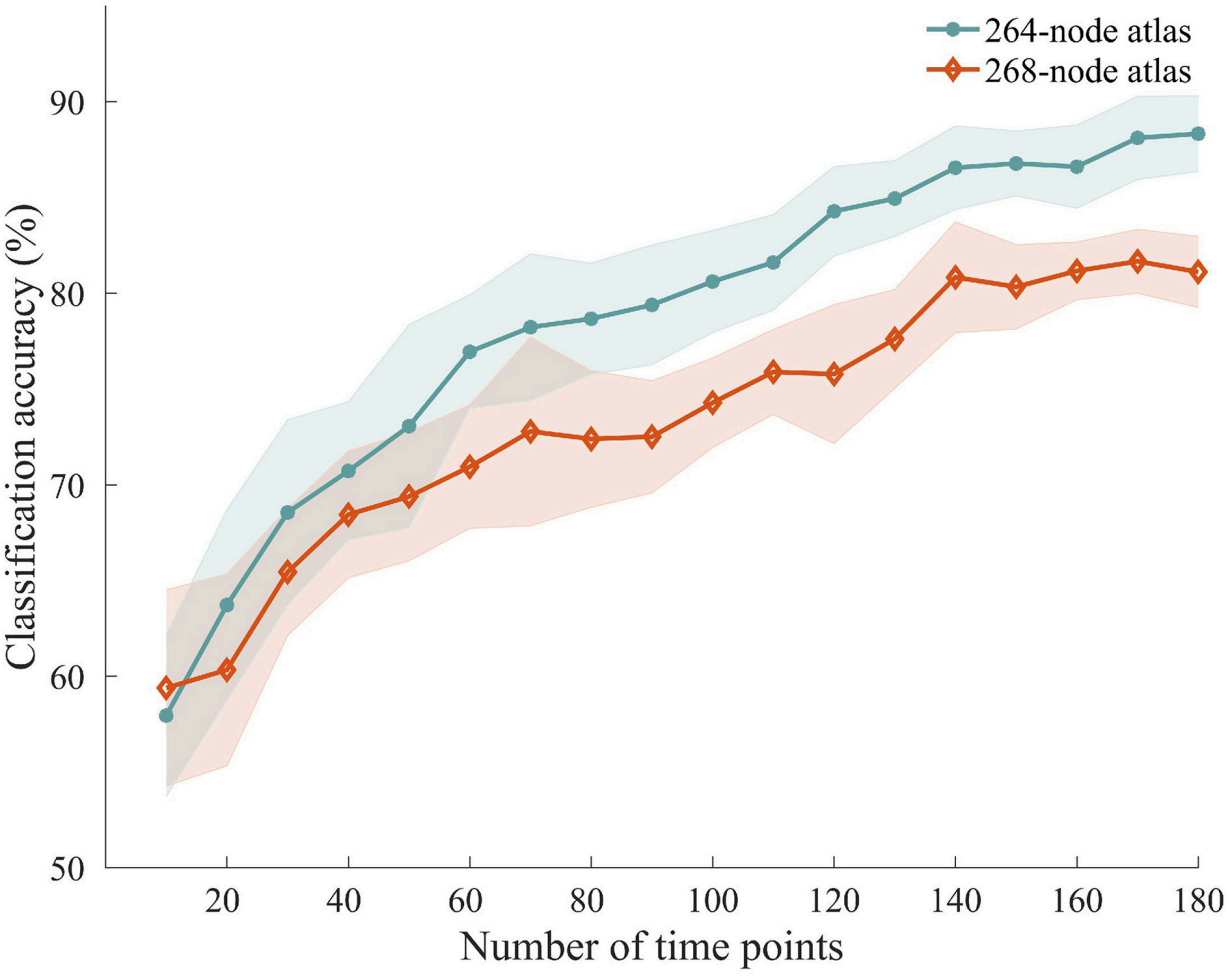
Classification accuracy using shorter time series. ISFC patterns were calculated with shorter time series and processed through the same classification procedure. Solid lines and shaded areas indicate the mean accuracies and SEMs across the 10 randomizations, respectively. Two different atlases were used for this analysis.

To explore how the number of time points used for ISFC estimation influenced the classification accuracy, we performed the classification with ISFC calculated using a varying number of time points between 10 and 180. We observed accuracies ranging from 57.94 to 88.33%, with higher accuracies obtained using larger numbers of time points (Figure 6). Permutation testing revealed that the accuracies were significantly higher than chance with scan lengths as short as 20 time points (40 s), suggesting that attentional modulation of ISFC can be reliably detected using relatively short scan lengths.

### 3.7. Effects of Atlas on Classification

We repeated the classification analyses using ISFC measures calculated from a 268-node atlas that divides the human brain into 8 networks. As expected, the subnetwork-based analysis revealed a large number of within-network and across-network ISFC measures that were discriminative in task classification (Figure 5B). In addition, the average classification accuracies found using across-network ISFC measures between a certain subnetwork and every other subnetwork were significantly higher than chance. For classifications using shorter time courses, the use of this 268-node atlas led to similar accuracies as the 264-node atlas, with accuracies ranging from 59.39 to 81.11% (Figure 6). These results suggest that our main findings have robust reproducibility.

### 3.8. Effects of Head Motion

Previous studies have suggested that head motion can influence the estimation of FC (Power et al., 2012; Van Dijk et al., 2012). However, motion artifacts are unlikely to have contributed to the observed successful task context classification using ISFC, given that the classification accuracy determined using classic FC measures was much lower than that of the ISFC measures. To remain conservative, however, we further examined the motion estimates for the two attention tasks. Using the motion parameters generated during the motion correction procedure during preprocessing, we calculated the average frame-to-frame motion for each experimental run and each subject (Power et al., 2012). This process resulted in a total of 60 (12 subjects, each performed 5 runs per attention task) values for each task. These values were assessed using paired t-tests to compare the head motion difference between the two attention tasks. We found that the difference between the two attention tasks was not significant (*t*_(59)_ = 1.568, *p* = 0.12). Therefore, the successful classification of task goals is unlikely to be based on motion artifacts.

## 4. Discussion

In this study, we investigated how behavioral goals modulate the whole-brain FC of subjects. In contrast to previous goal representation studies that used rudimentary stimuli designed for a laboratory setting, dynamic, complex naturalistic stimuli that conveyed rich information were used in this experiment. This naturalistic paradigm suitably mimicked goal-directed behavior in a real-life context. Given that top-down information processing recruits wide swaths of brain regions, we examined large-scale FC across the whole brain. We employed a novel ISFC method to isolate task-evoked FC from intrinsic neural fluctuations and non-neuronal artifacts. We first conducted a similarity analysis and showed that ISFC patterns were more efficient in representing specific task context than standard FC patterns. We then employed MVPA methods to examine whether attention tasks could be predicted from the corresponding ISFC patterns. We found that ISFC reliably distinguished one attention task from another with a high classification accuracy, even with relatively short scan lengths. We further identified many within-network and across-network ISFC measures that enabled task classification, suggesting a global modulation of connectivity patterns by task contexts.

Multivariate approaches ensure high sensitivity to fine-grained discriminative patterns (Norman et al., 2006; Zeng et al., 2012; Haxby et al., 2014), and recent MVPA research investigating task representation has shown that distributed patterns of activity in the parietal, medial and lateral prefrontal cortex (PFC) represent top-down tasks (goals) (Chiu et al., 2011; Waskom et al., 2014; Long and Kuhl, 2018). However, these findings were largely based on simple stimuli and task rules and overlooked the interactions between regions. Our results extend these studies by showing that selective processing of complex visual information conveyed by naturalistic stimuli modulated large-scale brain networks and that this modulation contained highly predictive information on the task contexts. We employed a LOSOCV procedure to estimate task classification performance. This across-subject MVPA can be challenging, given high levels of interindividual variability (Finn et al., 2015). For example, in a previous study with a LOSOCV setting, standard FC patterns successfully predicted which task a subject was performing, but the classification accuracies were relatively low (Cole et al., 2013). In the present study, the accuracy obtained using standard FC patterns was not significant, which is consistent with the similarity analysis showing that the standard FC patterns of the two tasks were highly correlated. This negative result may be attributed to the complex information contained in the naturalistic stimuli, which drives complex neural responses and thus hinders the detection of subtle differences between two tasks. On the other hand, the modulation of FC measures was possibly overwhelmed by intrinsic FC patterns, as previous studies have found that resting-state FC largely matches the FC during task performance (Cole et al., 2014; Kim et al., 2017). In contrast, we obtained a high level of accuracy when using ISFC patterns for the classification, and the accuracies remained significant even when very short time courses were used for ISFC pattern estimation. By repeating the MVPA procedure using another brain atlas, we have also shown that the performance was not specific to the choice of atlas. Along with previous ISFC studies (Simony et al., 2016; Kim et al., 2017; Rosenthal et al., 2017), the current successful classification of attention tasks shows promise for the utilization of the ISFC method in other contexts to investigate subtle differences between task-evoked FC patterns.

The edge-based analysis and subnetwork-based analysis found that ISFC measures within the visual network and between this network and many other networks largely contributed to task classification, indicating that connections with visual regions are extensively modulated by behavioral goals. The activity of visual regions is modulated in a variety of attention tasks, possibly reflects the differentiated representation of visual stimuli under specific task context (Mirabella et al., 2007; Reynolds and Heeger, 2009; Jehee et al., 2011). Recent MVPA studies have also found that activity in the visual cortex provides discriminative information on which visual dimension of a stimulus the subjects are processing (Waskom et al., 2014). In addition to biased neural activity, many neuroimaging studies have found that interactions with visual regions are modulated by behavioral goals (Maunsell and Treue, 2006; Al-Aidroos et al., 2012). Attention to different visual categories modulates connections between the occipital and ventral temporal cortexes (Al-Aidroos et al., 2012). And interactions between primary visual regions and frontoparietal regions were enhanced when visual stimuli were attended (Griffis et al., 2015). We provide additional evidence that connections within the visual network and across-network connections between the visual network and many other networks, including the dorsal attention, frontoparietal, and default mode networks, support the reliable discrimination of tasks under naturalistic conditions. Interactions between the frontoparietal and sensory regions are widely thought to play crucial roles in the biased processing of goal-relevant sensory information (Miller and Cohen, 2003; Vossel et al., 2014). Furthermore, enhancements in the connections between the dorsal attention network and the visual network have been observed during natural movie watching, with the possible function of controlling attention to the display (Kim et al., 2017). Our findings are well aligned with these studies. Although there is evidence that the biasing of sensory areas emerges from the frontoparietal regions (Bressler et al., 2008; Baldauf and Desimone, 2014), we cannot investigate this causal relationship because of the use of Pearson correlations to represent interactions between regions. Methods such as dynamic causal modeling (Friston et al., 2003) or Granger causality (Roebroeck et al., 2005) may be employed for future explorations of the direction of influences between regions.

The employed MVPA methods also revealed many other within- and across-network ISFC measures that were modulated by top-down goals. Indeed, we found that almost every canonical subnetwork formed discriminative connections with other subnetworks. Within-network connections in the dorsal attention network and across-network connections between the dorsal attention network and other networks such as the default mode, somatomotor, and frontoparietal networks resulted in high classification accuracies. Recent human neuroimaging experiments and studies in stroke patients have suggested that the dorsal attention network is largely involved in mediating the top-down guided voluntary allocation of attention to locations or features (Ptak and Schnider, 2010; Vossel et al., 2014). Regions from the dorsal attention and frontoparietal networks have also been consistently highlighted in task context representations (Chiu et al., 2011; Waskom et al., 2014; Long and Kuhl, 2018). Our MVPA results are consistent with these findings, suggesting that functional connections with frontoparietal regions are differentially modulated by behavioral goals. The successful task classification based on across-network connections with the somatomotor network may be due to the biased processing of action information in the behavioral attention task, since action observations activate the somatomotor regions (Buccino et al., 2001) and engage a network of sensorimotor brain regions called the action observation network (Gardner et al., 2015). Natural vision has been shown to modulate large-scale network interactions, and recent attention studies using naturalistic paradigms have demonstrated that attention to complex semantic information changes the neural activity of widely distributed regions (Cukur et al., 2013; Nastase et al., 2017). Our current findings of distinct changes in broadly distributed within-network and across-network connectivity suggest that goal-directed behavior under naturalistic conditions is reflected not solely by local changes in specific activations or connectivity but likely by systematic changes across large-scale brain networks. One limitation of this study is that the sample size of the publicly available dataset we used is relatively small (12 subjects). This relatively small sample size may compromise the reliability of experimental results to some degree. In the future, we should collect more fMRI data by ourselves to enhance the experimental results.

In summary, using the novel ISFC method, we show that selective processing of complex visual information under naturalistic conditions modulates large-scale FC and that this modulation supports the reliable discrimination of top-down task goals. We identified a large number of within-network and across-network discriminative connections, suggesting that goal-directed processing of naturalistic stimuli modulates the coordination of wide swaths of brain regions that belong to different canonical functional networks. This analysis based on large-scale brain networks extends previous studies of goal-directed behavior that focused on changes in local neural activity by showing that the modulation of connectivity between brain regions is broadly distributed. Our study may shed light on the role of large-scale brain networks in goal-directed behavior and suggests that the ISFC may provide an efficient method for identifying task-evoked networks.

## Ethics Statement

All participants provided written, informed consent prior to participating in the study in compliance with the Committee for the Protection of Human Subjects at Dartmouth College, including a provision for data to be shared with other researchers around the world or on a publicly available data archive. The study was approved by the Institutional Review Board of Dartmouth College, and participants received monetary compensation for their participation.

## Author Contributions

ZW and YL designed research and performed research. ZW, TY, XY, and YL analyzed data. ZW, TY, and YL wrote the paper.

### Conflict of Interest Statement

The authors declare that the research was conducted in the absence of any commercial or financial relationships that could be construed as a potential conflict of interest.
